# Brain MRI detection of early Wernicke’s encephalopathy in a hemodialysis patient

**DOI:** 10.1002/ccr3.5539

**Published:** 2022-03-03

**Authors:** Daiki Aomura, Yukifumi Kurasawa, Makoto Harada, Koji Hashimoto, Yuji Kamijo

**Affiliations:** ^1^ Department of Nephrology Shinshu University School of Medicine Matsumoto Japan; ^2^ Department of Health Promotion Medicine Shinshu University School of Medicine Matsumoto Japan; ^3^ Department of Internal Medicine Yodakubo Hospital Nagawa Japan

**Keywords:** appetite loss, brain magnetic resonance imaging, end‐stage renal disease, thiamine deficiency, wernicke's encephalopathy

## Abstract

A 93‐year‐old hemodialysis patient became hospitalized for thiamine deficiency‐induced appetite loss and ultimately experienced consciousness disorder diagnosed as Wernicke's encephalopathy (WE). Since brain MRI on admission was retrospectively found to display faint WE findings, careful brain MRI assessment is recommended in hemodialysis patients with appetite loss alone.

## Graphical abstract

Wernicke's encephalopathy should be suspected in hemodialysis patients with appetite loss alone, with careful brain MRI analysis for detecting possible Wernicke's encephalopathy at an early stage.

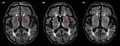

## 1 CASE

**FIGURE 1:**
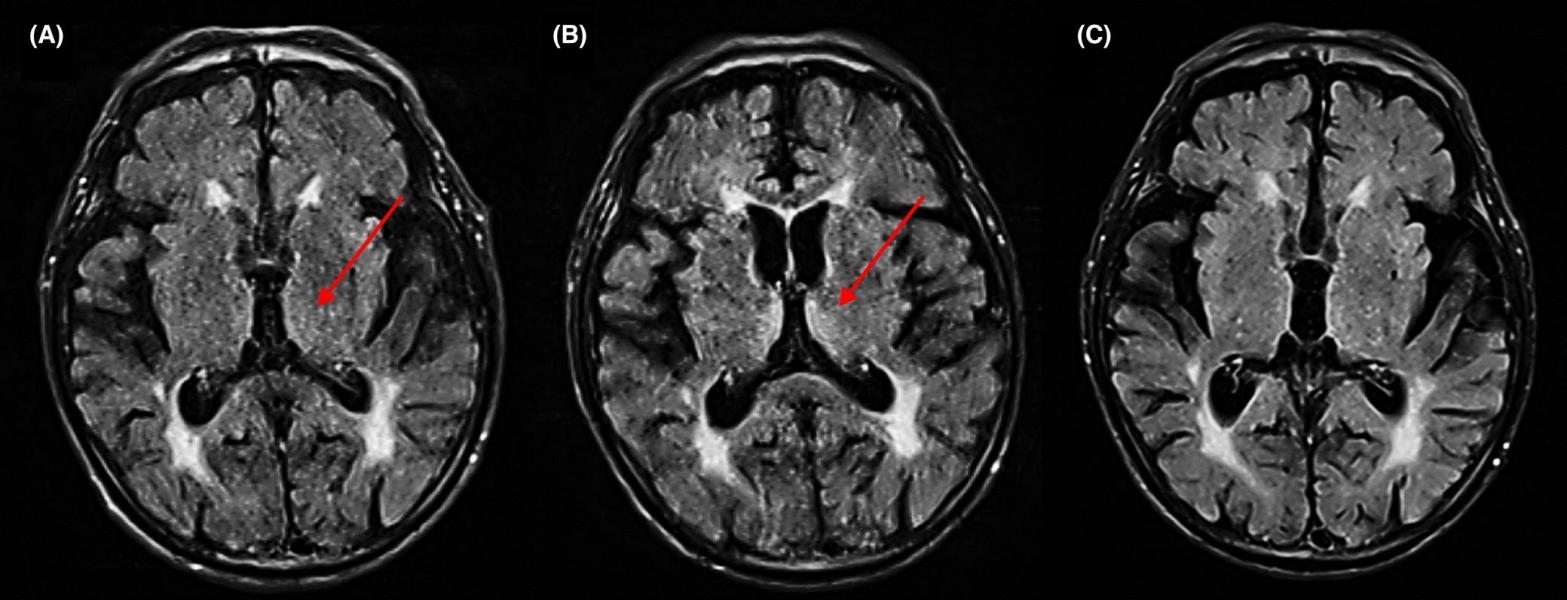
Findings of head magnetic resonance imaging with T2‐weighted fluid‐attenuated inversion recovery sequences (A) on admission with loss of appetite, (B) on the 14th day of admission with Wernicke's encephalopathy, and (C) at 1 month after thiamine injection. Red arrows indicate symmetric increased signals inside the thalami, a common and characteristic finding of Wernicke's encephalopathy

A 93‐year‐old male hemodialysis patient with no alcohol consumption was hospitalized complaining of gradual appetite loss, muscle weakness, and falls. He had hypertension and nephrosclerosis and was taking anti‐hypertensives. Various screening tests, including brain magnetic resonance imaging (MRI) to exclude central nervous disorders, could not identify the cause of his symptoms. Fourteen days after, he suffered a decreased consciousness, and ensuing MRI revealed findings of Wernicke's encephalopathy (WE) inside the thalami (Figure 1B). Retrospective analysis of the MRI on admission also showed slight signals in the same region (Figure 1A). Thiamine injection fully restored his consciousness, MRI findings (Figure 1C), and appetite without sequelae, implicating appetite loss as the initial WE symptom. Considering the significant thiamine removal during hemodialysis,^1^ his WE was presumably caused by reduced food intake and hemodialysis, and then worsened by WE itself. The non‐specific symptoms of WE make early diagnosis difficult,^2^ with few studies on its initial course. The present report could follow the entire progression of WE, revealing the presence of brain MRI findings even at the time of appetite loss. WE should be suspected in hemodialysis patients with appetite loss alone, with careful brain MRI analysis for detecting possible WE at an early stage.

## CONFLICT OF INTEREST

No conflicts of interests.

## AUTHOR CONTRIBUTION

D.A. drafted the article. Y.K., M.H., K.H., and Y.K. revised the article critically for important intellectual content and gave final approval of the version to be submitted.

## ETHICAL APPROVAL

The present case report adhered to the Declaration of Helsinki.

## CONSENT

Patient consent has been signed and collected in accordance with the journal's patient consent policy.

## Data Availability

Data sharing not applicable to this article as no datasets were generated or analysed during the current study.
